# Computed tomography-based angle measurements of the sagittal capitulum and trochlea position in relation to the humeral shaft

**DOI:** 10.1007/s00276-023-03118-7

**Published:** 2023-03-09

**Authors:** Axel Przyklenk, Michael Hackl, Andra-Iza Iuga, Tim Leschinger, David Maintz, Andreas Harbrecht, Lars Peter Müller, Kilian Wegmann

**Affiliations:** 1 Department of Orthopedic and Trauma Surgery, University of Cologne, Faculty of Medicine and University Hospital Cologne, Cologne, Germany; 2 Institute of Diagnostic and Interventional Radiology, University of Cologne, Faculty of Medicine and University Hospital Cologne, Cologne, Germany

**Keywords:** Articular surface angulation, Computed tomography, Distal humerus, Elbow, Skeletal radiology

## Abstract

The radiologic evaluation of the sagittal angulation of the distal humerus is commonly based on standard lateral radiographs. However, lateral radiographs do not allow to examine the lateral angulation of the capitulum and the trochlea, separately. Although this problem could be approached via computed tomography, there are no data available describing the difference between the angulation of the capitulum and trochlea. Therefore, we aimed to assess sagittal angles of the capitulum and trochlea in relation to the humeral shaft based on 400 CT-scans of the elbow in healthy adults. Angles were measured in sagittal planes at the capitulum center and three anatomically defined trochlea locations and were spanned between the axis of the joint component and the humerus shaft. Angles were tested for differences between measurement locations and correlation with patient characteristics (age, sex, trans-epicondylar distance). Angles increased from lateral to medial measurement locations (107.4 ± 9.6°, 167.4 ± 8.2°, 171.8 ± 7.3°, 179.1 ± 7.0°; *p* < 0.05). Largest angle differences were detected between the capitulum and trochlea with smallest angles measured at the capitulum. Patient characteristics did not correlate with angles (*p* > 0.05). Intra-rater-reliability was *r* = 0.79–0.86. As CT-imaging allows to distinguish between sagittal capitulum and trochlea locations, it might benefit the radiologic diagnostic of sagittal malalignments of the distal humerus at the capitulum and trochlea, separately.

## Introduction

The anatomy of the elbow is known to be complex [1–5]. In the clinical setting, the radiologic evaluation of the anatomy of the distal humerus is applied on standard radiographs as first-line imaging to assess the bone for fracture signs and malalignment [6–8]. Following standard radiographs of the distal humerus, computed tomography (CT) is frequently used as it allows a more detailed imaging of osseous structures [6, 9, 10]. However, it is not used to assess the alignment of the distal humerus through standardized methods so far.

Instead, the alignment of the distal humerus is traditionally described in sagittal standard radiographs [6, 11–14]. One method to detect sagittal malformations of the distal humerus is the application of the anterior humeral line which is described to intersect the capitulum in its middle third [6, 14]. Yet, it was criticized that a shift of the intersection of the anterior humeral line with the capitulum might primarily detect translational deformities [8, 15]. Therefore, it is suggested to consider sagittal angle measurements for the radiographic examination of the distal humerus [8]. A prominent method to assess sagittal angles at the distal humerus uses the angular difference between the longitudinal humerus shaft axis and an axis bisecting the capitulum [11]. Although this method solely measures the angulation of the capitulum with no regard to the trochlea it is commonly stated as the sagittal angulation of the condyles, joint component or joint block [11]. Applied on male and female children as well as adults, condyles were described to be 30–40° anteriorly angled in lateral radiographs of the healthy elbow [11, 16] which is equivalent to sagittal angles of 140–150° spanned between the sagittal humeral shaft and the condyle axis. However, when examining the standard lateral radiograph of the elbow it can be suggested that the angle between the trochlea and the humeral shaft is greater compared to the angle at capitulum level [11]. Unfortunately, the standard radiograph does not allow precise isolated measurements at different locations at capitulum as well as trochlea level due to superimposition. In turn, by applying cross-sectional CT imaging, different locations at the trochlea and the capitulum in relation to the humeral shaft are made accessible. Although sagittal angle measurements of the distal humerus based on CT-imaging would enable a more precise assessment of the alignment of the distal humerus, there are no reported studies investigating this issue. Thus, no standard values describing the anterior angulation of the trochlea are available in the literature.

Therefore, we aim to assess the sagittal angles of the capitulum and the trochlea in relation to the humeral shaft at different locations by applying a standardized measurement protocol on native multi-planar reconstructed CT-scans of the elbow in healthy adults. We further aim to test for correlation between patient characteristics (age, sex and trans-epicondylar distance [TED]) and angles. We hypothesized that the sagittal angle between the trochlea and the humerus shaft is significantly greater compared to the sagittal angle between the capitulum and the humerus shaft and that angle do not correlate with age, sex and TED.

## Materials and methods

This retrospective analysis was performed on native multi-planar reconstructed CT-scans of the elbow of patients differing in age and sex.

The measurement protocol was developed by experienced trauma surgeons and revised by experienced radiologists of our institution. Measurements were conducted by a medical doctoral candidate as member of the trauma surgery department trained in skeletal radiology. Besides age and sex, the interpreter had no information about the medical history of the patients enrolled in this study.

Approval from the Institutional Review Board was obtained (21–1597-retro) and in keeping with the policies for a retrospective study, informed consent was not required. The participants’ anonymity is preserved throughout this study.

### CT-scan

**Fig. 1:**
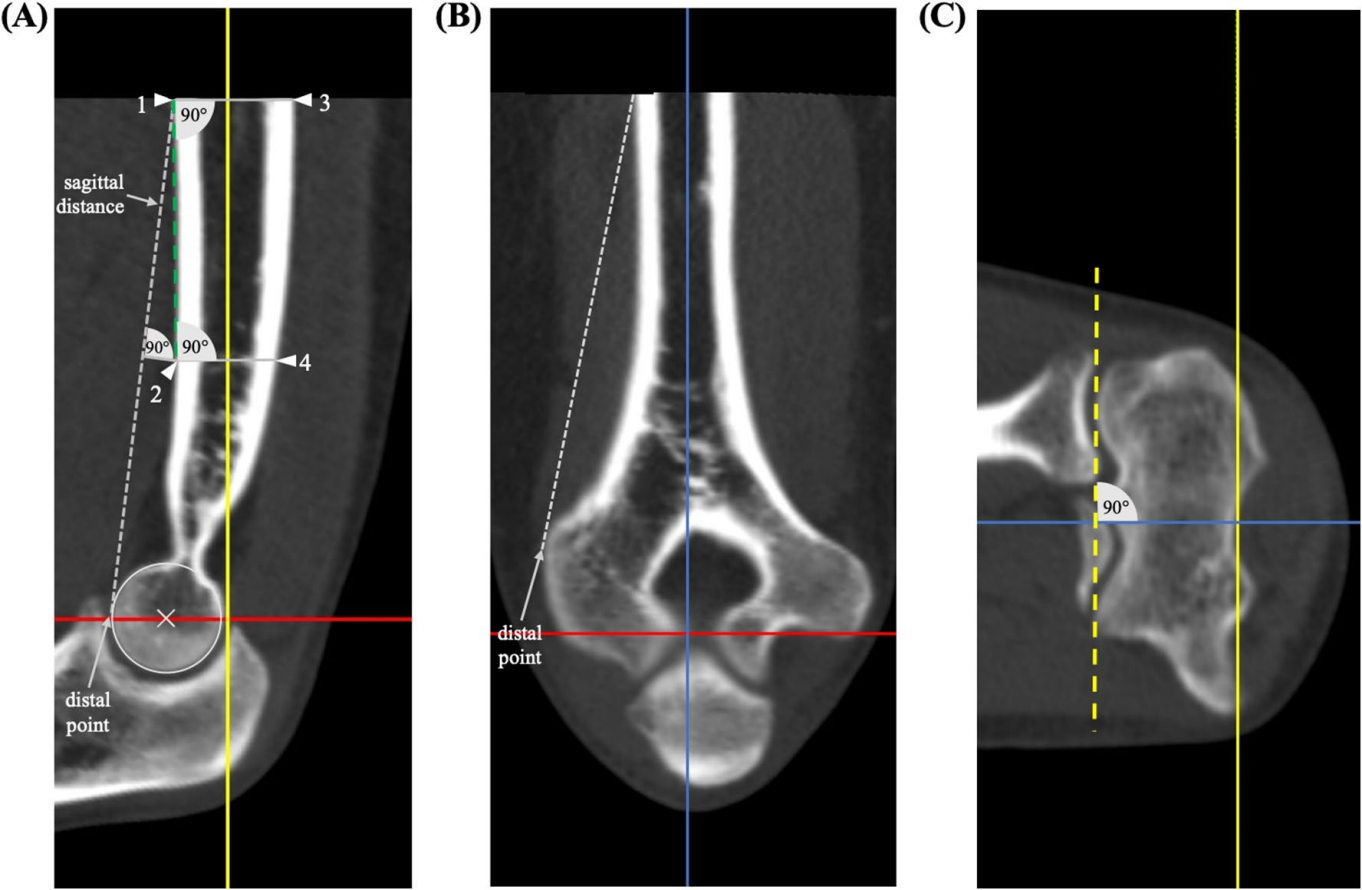
CT standard planes and determination of humeral shaft axis. The determination of the humerus shaft axis as applied for defining the standard planes is described in the section ‘humerus shaft axis’ and is depicted in this figure (**A**). The determination of the coronal humerus shaft axis (**B**) is achieved via the same approach as described for the sagittal view. The ‘distal point’ is defined as the most volar location on the trochlea in the sagittal view and the most lateral location on the lateral epicondyle in the coronal view and serves for determination of the sagittal distance. Red line: axial plane, bisects the trochlea in the sagittal view. Yellow line: coronal plane, humerus shaft axis in the sagittal view and parallel to the joint surface (dashed yellow line) in the axial view (C). Blue line: sagittal plane, humerus shaft axis in the coronal view. Grey dashed line: sagittal distance. Green dashed line: connection of point 1 and 2. Circle: trochlea surface (color figure online)

400 CT-scans were randomly enrolled in accordance with the following inclusion criteria: epiphyseal closure, no direct fracture signs or implants at the distal humerus as well as a CT slice thickness ≤ 1.0 mm. The CT imaging was performed between 2011 and 2016. CT-scans of the elbow were performed with clinical indications apart from our study. For example, when patients presented clinical symptoms that indicated osseous damage following adequate trauma to the elbow (e.g., luxation, fall, polytrauma) that could not be excluded via standard radiographs. Only CT scans of elbows that experienced radiologists found to show no signs of osseous damage at the distal humerus were enrolled in our investigations. The CT-technique used 120 kVP for all scans and a varying slice thickness of 0.84 mm ± 0.1 mm (mean ± standard deviation), 126.6 mAs ± 29.6 mAs, a field of view of 180.0 mm ± 56.7 mm, pixel size of 0.25 mm ± 0.1 mm and a pitch of 0.43 ± 0.2. Only CT scans with a sagittal distance (Fig. 1) greater than 5 mm were enrolled in our investigations. Average sagittal distance was 72.6 mm ± 16.8 mm.

### Study design

400 CT-scans were analyzed following the standardized measurement protocol below. Subsequently, to calculated intra-rater-reliability, 100 randomly selected CT-scans of the total 400 were analyzed again following the same protocol after a period of 2 weeks, where no measurements were conducted.

### Measurement protocol

This protocol was developed to perform a standardized angle determination between the axes through the spherical joint component of the distal humerus and the center of the humerus stem. Angle measurement was conducted in the sagittal planes of multi-planar (axial, coronal, sagittal) reconstructed CT-scans. The three orthogonal-related planes were adjusted in a standardized manner. The sagittal planes being measured, were selected in the axial plane. All measurements were performed using IMPAX EE software (AGFA Health Care, Mortsel, Belgium). The average time for one measurement including the adjustment of the standard planes and measurements of all angles was 4 min 47 s ± 1 min 41 s.

### CT standard planes

The coronal plane and is adjusted parallel to the joint line in the axial view (Fig. 1C) and as a bisecting line of the trochlea in the sagittal view (Fig. 1A). In the sagittal view (Fig. 1A), the coronal plane parallels the longitudinal axis of the humerus shaft. The sagittal plane is adjusted parallel to the longitudinal axis of the humerus shaft in the coronal view (Fig. 1B). In the axial view, the coronal and sagittal plane are orthogonal to each other, whereas in the coronal view, the axial and sagittal plane and in the sagittal view, the coronal and axial plane are orthogonal to each other.

### Humerus shaft axis

The humerus shaft axis was defined using a ‘centerline by four points’ in the sagittal and coronal view using IMPAX EE software (AGFA Health Care, Mortsel, Belgium). The four points were adjusted according to a standardized method (Fig. 1A).

Sagittal view: Point 1 was defined as the most proximal point on the volar outer cortical surface of the humerus shaft. Point 2 was adjusted perpendicular to the middle of the sagittal distance which was defined as the distance between point 1 and the most volar point on the trochlea (distal point). Point 3 was adjusted on the dorsal outer cortical surface opposite to point 1 and perpendicular to a connecting line between point 1 and point 2. Point 4 was adjusted on the dorsal outer cortical surface opposite to point 2 and perpendicular to a connecting line between point 1 and point 2.

Coronal view: Point 1 was defined as the most proximal point on the lateral outer cortical surface of the humerus shaft. Point 2 was adjusted perpendicular to the middle of the distance between point 1 and the most lateral point of the lateral epicondyle (distal point). Point 3 and point 4 were located on the medial outer cortical surface of the humerus and were adjusted as described for the sagittal view.

The sagittal distance was restricted to a minimum of 50 mm and maximum of 100 mm to standardize the adjustment of the humerus shaft axis and to be able to use CT images that focused primarily on the elbow. CT images depicting a sagittal distance < 50 mm were excluded. When the sagittal distance was > 100 mm, point 1 was adjusted to a more distal location resulting in a sagittal distance of 100 mm.

### Measurement locations

**Fig. 2:**
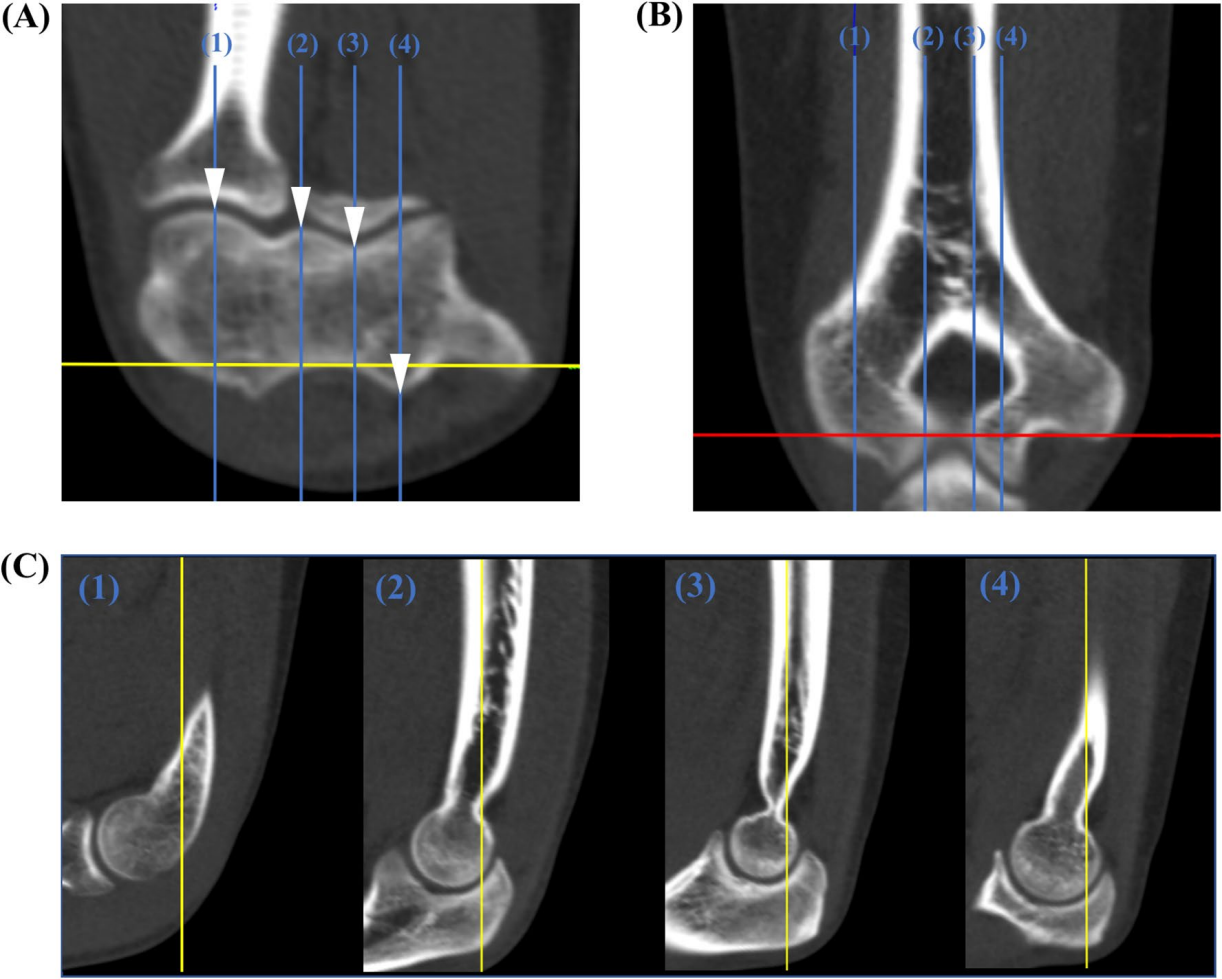
Angle measurement locations. Anatomical landmarks for definition of measurement locations are marked by white arrows in the axial view (**A**). Sagittal plane positioning (blue line) as applied for the angle measurement is shown for each measurement location (1–4) in the axial view with the corresponding location in the coronal view (**B**). Angle measurements are conducted in the sagittal view (**C**) in relation to the longitudinal humerus shaft axis (coronal plane: yellow line) which is depicted for each measurement location (1–4). Red line: axial plane (color figure online)

Sagittal measurement slices for angle determination were selected by positioning the sagittal plane in the axial view on defined bone landmarks at the distal humerus. The landmarks serving as measurement locations (Fig. 2), were 1) the center of the capitulum humeri, 2) the volar edge of the lateral trochlea lip, 3) the volar part of the trochlea groove and 4) the dorsal edge of the medial trochlea lip.

### Angle measurement

**Fig. 3:**
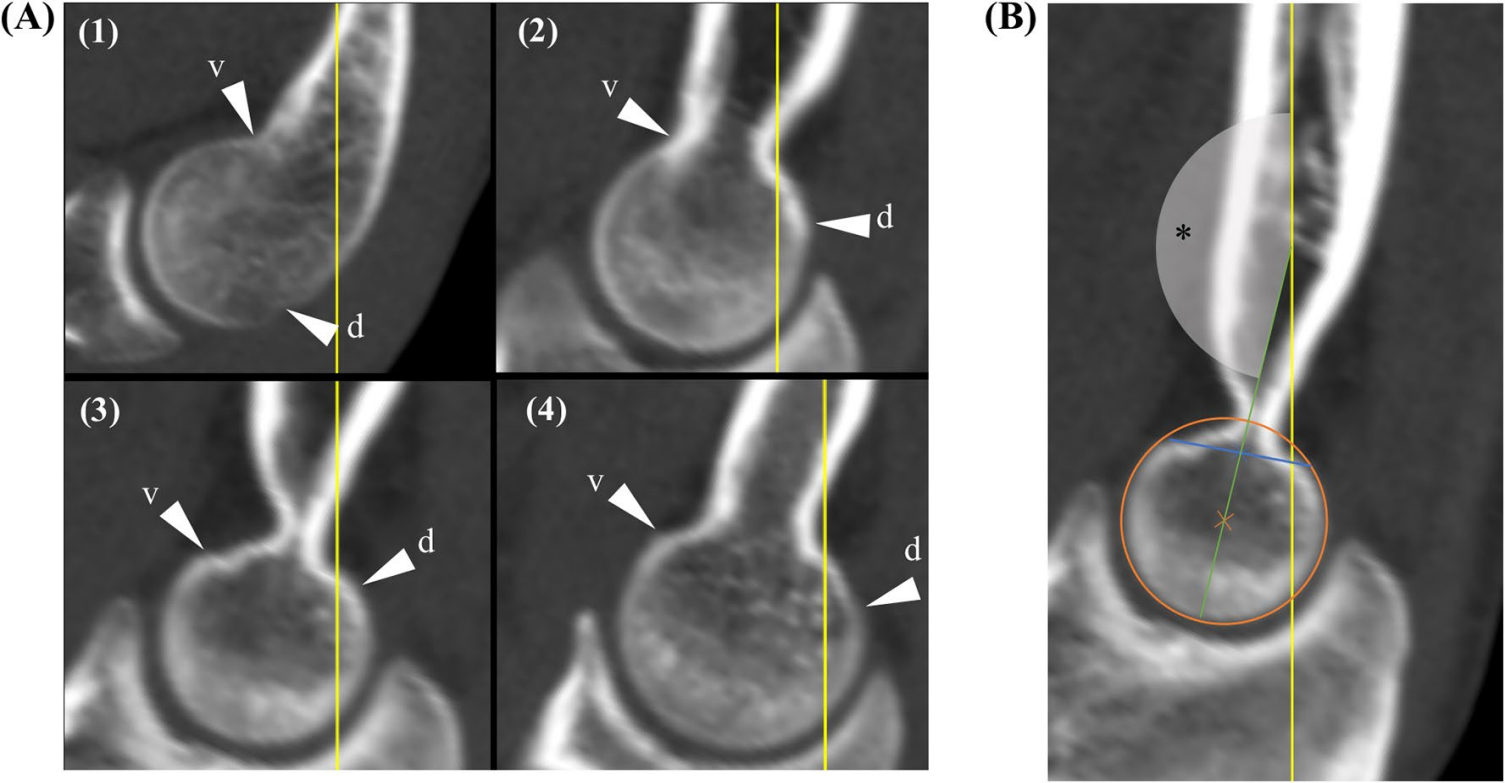
Angle measurement. At first, the volar (v) and dorsal (d) transmission from a convex to a lineal or concave surface of the spherical joint component was determined (white arrow) (**A**) and is shown for each measurement location in the sagittal view (1–4). For measurement (**B**) the transmission points are connected (blue line) and a circle (orange circle) is inserted that matches the surface of the spherical joint component. The angle (*) is measured between the longitudinal shaft axis (yellow line) and a line (green line) that connects the middle of the circle and the transmission point connecting line (blue line). Here, measurement location 3 (the volar part of the trochlea groove) was used for demonstration with an angle of 166°. These images are segments of the images depicted in Fig. 1C. For a wider depiction of these images, see Fig. 1C (measurement locations 1–4) (color figure online)

Angle determination was conducted in three steps. First, the distance between the dorsal and volar transition from a convex to a lineal or concave surface of the spherical joint component was determined (Fig. 3A, B). Second, a circle that corresponds to the spherical surface of the joint component was inserted (Fig. 3B). Third, the angle measurement was performed between the longitudinal axis of the humerus and an axis build by connecting the middle of the distance of the lineo/concavo-convex transitions (first step) with the middle of the circle (second step) (Fig. 3B). Values derived from angle measurements are stated in angular degrees (deg °).

When the determination of the lineo/concavo-convex transitions occur to be difficult, we recommend to first insert the circle which was described above as the second step and to consider the divergence of the trochlea /capitulum from the circle as the lineo/concavo-convex transitions. This was experienced to increase the accuracy especially for trochlear measurement locations when the lineo/concavo-convex transitions appeared vague (Fig. 3A).

### Trans-epicondylar distance (TED)

TED was determined in the coronal view. The TED was defined as the maximal distance between the outer cortical surface of the radial and ulnar epicondyles of the humerus [13]. TED is stated in millimeters (mm).

### Statistical methods

All analyses were performed using SPSS software (version 14; IBM, Chicago, Illinois). Normal distribution was tested using the Shapiro–Wilk test for each data set. Angle value differences between measurement locations were calculated conducting a repeated measure one-way analysis of variance (ANOVA). Angle value differences at the different measurement locations between males and female were tested via an ordinary two-way ANOVA. When the ANOVA was significant, post-hoc-testing was applied using the Bonferroni test for multiple comparisons. Age and TED-differences were tested via a Mann–Whitney *U* test. For correlation analysis—including correlation between patient characteristics (TED and age) and angle values as well as intra-rater-reliability the Spearman-correlation-coefficient r was computed. Correlation between patient characteristics (TED and age) and angles were considered perfect with Spearman-correlation-coefficient value r of + 1 or − 1, very strong for 0.8–0.9/− 0.8 to − 0.9, moderate for 0.6–0.7/− 0.6 to − 0.7, fair for 0.3–0.5/0.3 to − 0.5, poor for 0.1–0.2/− 0.1 to -0.2, none for *r* = 0. Reliability was considered excellent with *r* values of 0.8–1.0, good for 0.6–0.8, moderate for 0.4–0.6 and fair for 0.2–0.4, and poor for 0.0–0.2. The eta-coefficient was computed to investigate the association between the patients’ sex and angle values. Eta-coefficient values range between 0 and 1 and are interpreted-like Spearman-correlation-coefficient *r*. For each test, significance was assumed at *p* < 0.05*.*

## Results

### Descriptive data

**Fig. 4:**
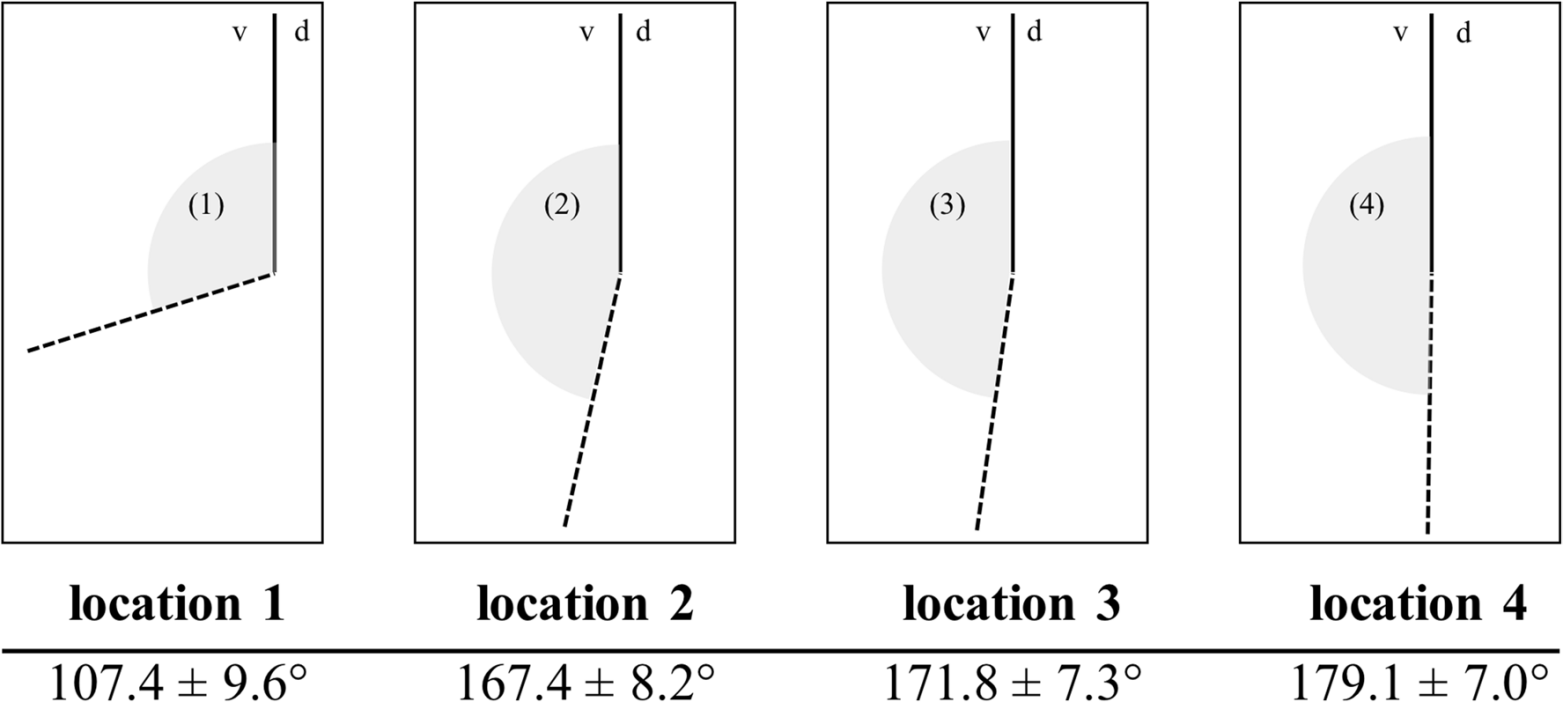
Angle values at measurement locations. Mean angles at each measurement location (1–4) are depicted in the sagittal view between the axis of the distal joint block (dotted line) and the longitudinal humeral shaft axis (solid line). Mean angle ± SD at each measurement location is stated below the corresponding depiction. d: dorsal, v: volar, °: degrees°. Measurement locations are depicted in Fig. 1

Data of patient characteristics (Table 1) and angles at the four described measurement locations are shown below (Fig. 4).

**Table 1:** Patient characteristics

	Overall (*n* = 400)	Male (*n* = 239)	Female (*n* = 161)
Age [yrs]	44.3 ± 16.3	42.1 ± 14.5	47.5 ± 18.3
TED [mm]	57.1 ± 6.0	60.7 ± 4.2	51.1 ± 4.1

*yrs* years; *mm* millimeter; *TED* trans-epicondylar distance, Mean ± standard deviation

### Data distribution

Normal distribution was calculated for the measured angles at each measurement location (*p* > 0.05). In addition, when data were analyzed in separated groups defined by sex, angles were normally distributed at each measurement location for both male and female (*p* > 0.05). Age and TED were not normally distributed, regardless if data were separated into sex-specific groups or not (*p* < 0.05). When 100 CT-scans were randomly selected to be measured a second time, the included angles derived from the first measurement at measurement location 1 were not normally distributed (*p* < 0.05).

### Angle measurements

**Fig. 5:**
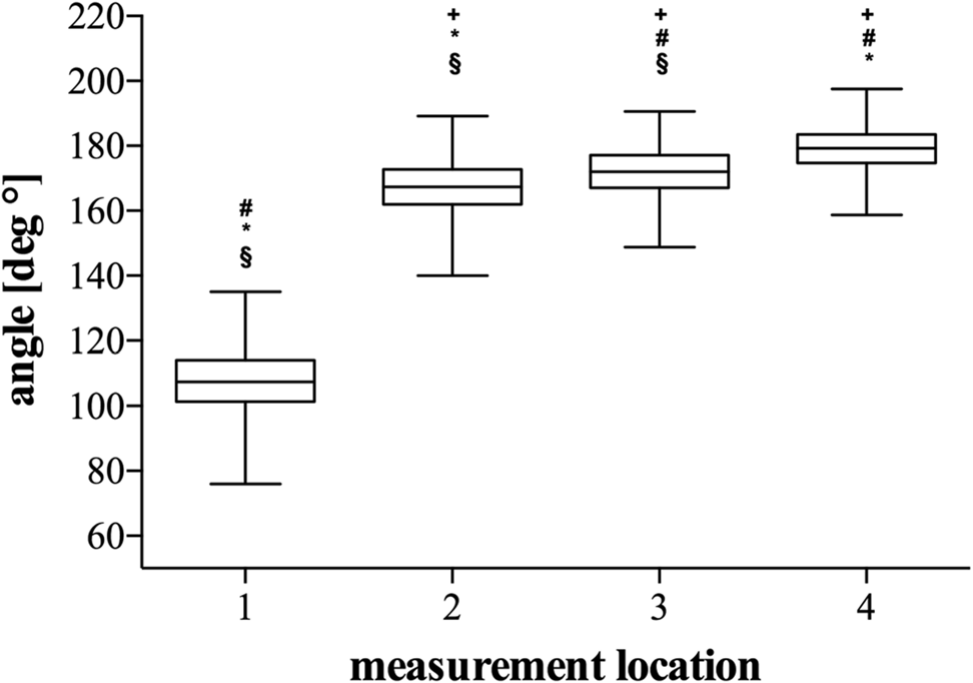
Results of overall angle measurements. Box–Whisker-Plots depicting the angles at each measurement location including median, upper and lower quartile as well as minimum and maximum, outliers are not shown. + : significant difference vs. location 1; #: significant difference vs. location 2. *: significant difference vs. location 3. §: significant difference vs. location 4; *p* < 0.05. Measurement locations are depicted in Fig. 1

The measured angles differed significantly (*p* < 0.05) between all measurement locations, while the angle degrees were detected to be the lowest at measurement location 1 and to be progressing from measurement location 1 to measurement location 4 (Figs. 4, 5).

### Sex-specific results

**Fig. 6:**
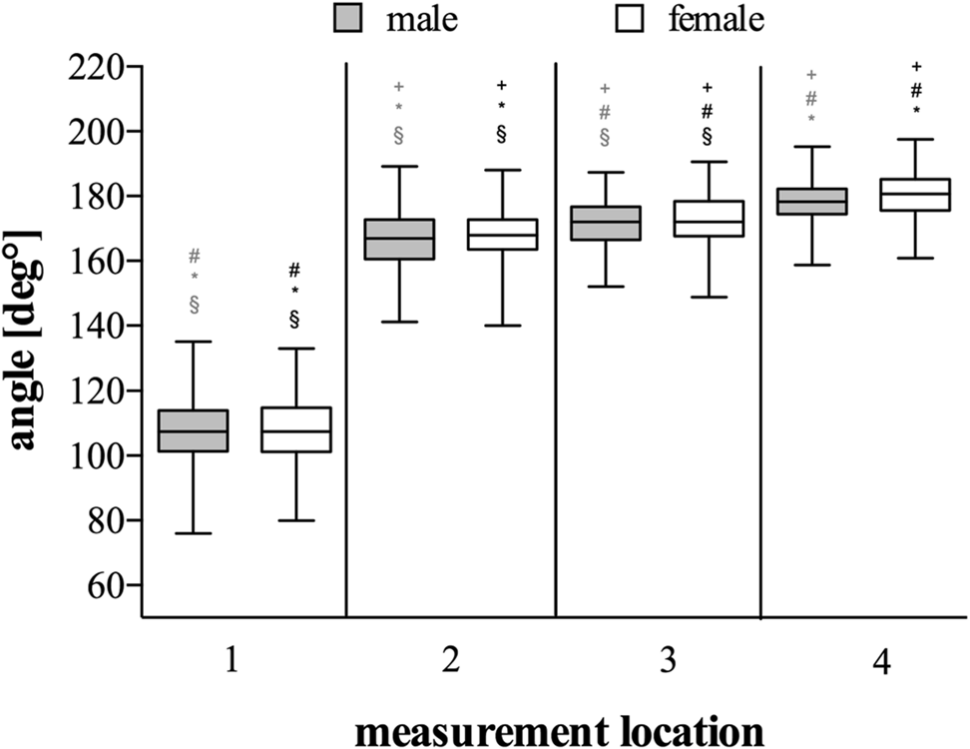
Results of sex-specific angle measurements. Box–Whisker-Plots depicting the angles at each measurement location in males (grey box) and females (white box) including median, upper and lower quartile as well as minimum and maximum, outliers are not shown. Grey signs indicate a significant difference vs. other location in males. Black signs indicate a significant difference vs. other location in females. ^+^: significant difference vs. location 1; ^#^: significant difference vs. location 2. ^*^: significant difference vs. location 3. ^§^: significant difference vs. location 4; *p* < 0.05. Measurement locations are depicted in Fig. 1

The females were significantly older (*p* < 0.05), while the TED was significantly shorter (*p* < 0.05) in females compared to males (Table 1). There were no differences (*p* > 0.05) regarding the angle degrees at all angle measurement locations between males and females (Fig. 6). Within both groups, angles were significantly different from another, while the means did not differ from the overall (non-sex-specific) angle measurement.

### Correlation/association analysis

Age and TED did not correlate with angle values. There was no association between sex and angles (eta = 0.03 to 0.1, *p* > 0.05) but a high association between sex and TED (eta = 0.97, *p* < 0.05) which was is in line with the significantly shorter TED in females compared to males reported above.

### Intra-rater-reliability

**Fig. 7:**
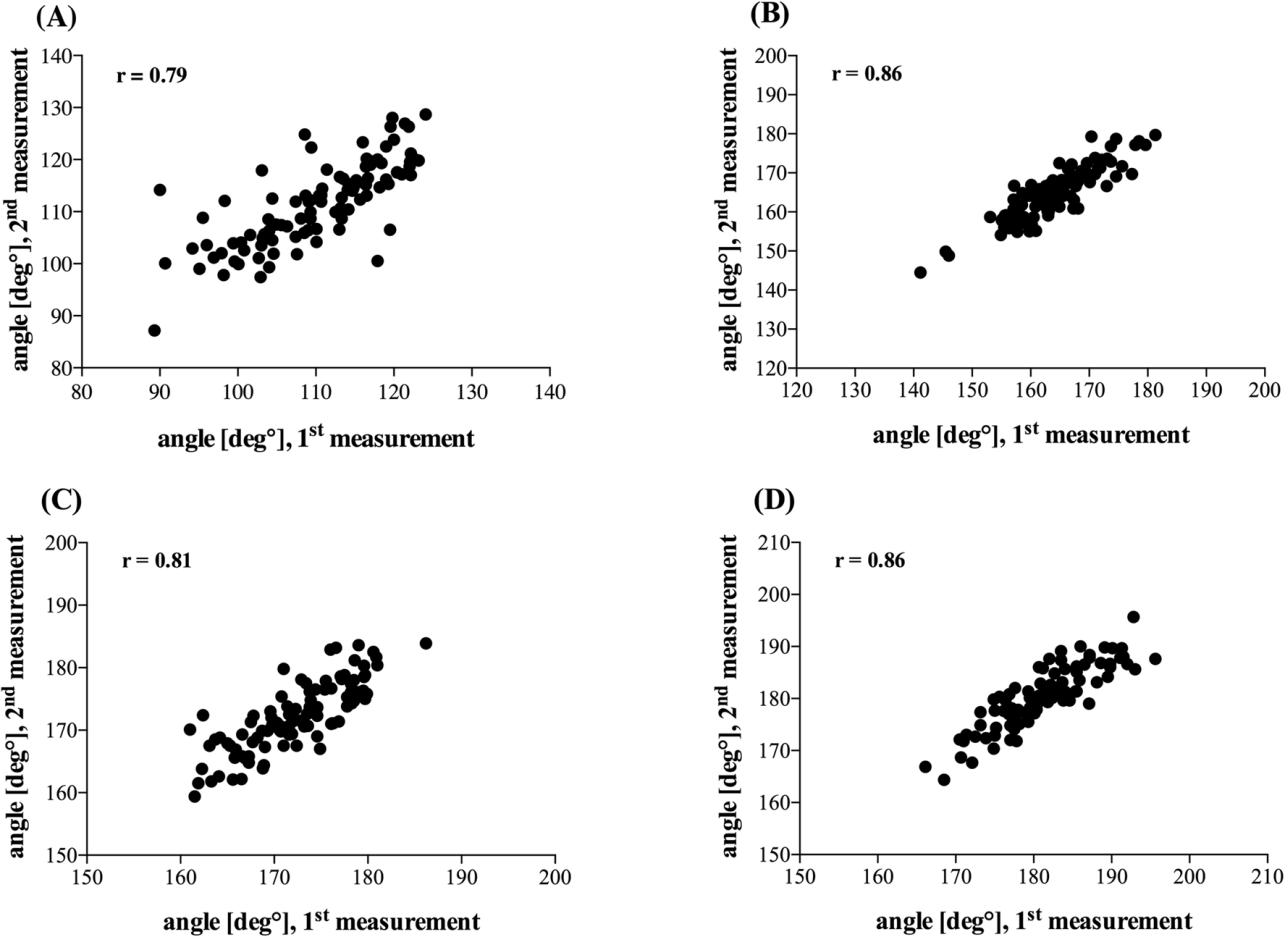
Intra-rater-reliability. Correlation of the first and second angle measurements at measurement location 1 (**A**), location 2 (**B**), location 3 (**C**), location 4 (**D**). *r*: Spearman correlation coefficient; *n* = 100

When measurements were repeated for the calculation of intra-rater-reliability, the angle values of the first and second measurement correlated significantly (*p* < 0.05) for all measurement locations (Fig. 7A-–D) and were accompanied by Spearman-coefficients from *r* = 0.79 to *r* = 0.86. Thus, intra-rater-reliability was proven to be good to excellent [10].

## Discussion

The assessment of the sagittal distal humerus alignment applying angle measurements between the articular surface and the humerus shaft is commonly performed on standard lateral radiographs [11]. Due to the complex anatomy of the distal humerus, particularly with respect to the difference in shape between the capitulum and trochlea [1], a precise imaging of these components is of special interest. CT-based sagittal angle measurements at different locations along the distal joint component may serve a more precise clinical diagnostic of elbow impairment and might improve the planning and evaluation of osteosynthesis procedures at the distal humerus. Although CT-imaging allows the evaluation of the capitulum and the trochlea at thin image slices, the difference in the sagittal angle between different locations along the capitulum and trochlea in relation to the humeral shaft are not described in the literature.

Hence, our study is the first to apply angle measurements in the sagittal plane of CT-scans of the distal humerus. Here, we show that the sagittal angles between the joint component and the humeral shaft differ depending on the location of measurement. We showed that the angle between capitulum center and humeral shaft is remarkably smaller compared to each angle at the trochlea. While the angles between the trochlea and humeral shaft—measured at the medial trochlear lip, the trochlear groove and the lateral trochlear lip—are close to another, it could be shown that these angles still increased from radial to ulnar slices differing significantly from another.

Considering the highly differentiated shape of the trochlea in the coronal view including a discrete lateral and a prominent more distal medial trochlea lip separated by the trochlea groove [1], our data add another aspect to the complex anatomy of the trochlea.

Also, as the elbow joint is presumably the most complex joint in the human body a differentiated radiologic approach was urgently needed allowing to consider the anatomical details of the capitulum and the trochlea as two major parts of the elbow joint. Via the articulation of the capitulum and the trochlea with the proximal radius and ulnar, respectively—two of the three joints of the elbow are built that allow flexion/extension (ulnohumeral joint), axial rotation and pivoting (radiocapitellar joint) [1]. While the shape of the capitulum (spherical) and trochlea (spool-like) itself dictates a high portion of the range of motion of the respective joint, the native sagittal position of the joint surface is essential to allow the optimal force development of contributing muscles upon elbow movement [1]. The sagittal position of the joint surface is described to be 30–40° anterior angulated which is assumed as the native alignment of the distal joint surface in relation to the humeral shaft allowing the physiologic range of the motion of the elbow [1, 11, 16]. When translating the angles of our study at capitulum center to sagittal angulation values, we show a larger anterior angulation (73°) compared to the literature (30–40°) [11]. Hence, the sagittal angle between capitulum and humeral shaft at the capitulum center of our study was sharper compared to the literature. This angle difference is presumably due to a difference in methods. In the literature, the axes for measurements are defined by the longitudinal humeral shaft axis and an axis bisecting the capitulum [11, 16]. We also used the humeral shaft axis but to increase reliability of our method we aimed to precisely consider the anatomical shape of the surface of the capitulum in the definition of the capitulum axis. We interpreted the line between the volar and dorsal transition from a convex to a linear or even concave shape of the capitulum surface in the sagittal view as the basis of the spherical part of the capitulum. Thereby, our method might have resulted in a greater angulation compared to values derived from lateral radiographs using a bisecting line through the capitulum. However, by applying these anatomical characteristics, the intra-rater-reliability of our measurements was good, almost excellent (*r* = 0.79), compared to a rather poor reliability (*r* = 0.39) as described in the literature when capitulum axis was defined differently in plain radiographs [11]. Although we maximized the standardization of our measurement, the average time for the measurement of one CT including all four measurement locations and the adjustment of the standard planes was only around 5 min (4 min 47 s ± 1 min 41 s). While this time could be reached in our final measurements, the average time for one measurement was rather longer than 10 min when we first tested our method. Therefore, the observer must be trained adequately to reach both high accuracy and short measurement time.

Knowing about the flaws of sagittal angle measurements in plain radiographs, such as the influence of expertise as well as often poor quality of images [8], we aimed to include only precisely reproducible osseous landmarks in our method. Thus, for defining the measurement locations along the trochlea, we considered prominent anatomical landmarks, such as the medial and lateral trochlea lip as well as the trochlea groove [1]. Proving our approach, the intra-rater-reliability for angle measurements was excellent (*r* > 0.8) at all trochlea locations.

Thereby, our approach enabled the assessment of the sagittal angle between the trochlea and the humeral shaft at different locations along the trochlea. Using CT-scans, we showed that the trochlea angulates significantly less anteriorly compared to the capitulum. While the angle between trochlea and humeral shaft was 167° at the lateral lip (measurement location 2) it increased to 171° at the trochlea groove (location 3) and ultimately to 179° at the medial lip (location 4) (Figs. 4, 5). Hence, the anterior angulation is shown to decrease as the trochlea even angulates posteriorly at the medial lip in some cases. This finding is of special importance as it is reasonable that the trochlea position in articulation with the proximal ulna contributes a major limitation to flexion/extension when the trochlea is misaligned post-surgically or genetically.

Thus, our data show, that the established normal values of 30–40° anterior angulation of the articular surface or also known as the joint block in relation to the humeral shaft do not match the unique angulation of the capitulum and trochlea, respectively. This underlines the enormous potential of CT-imaging in the evaluation of the alignment of the distal humerus considering the different sagittal position of the trochlea and the capitulum.

## Limitations

One major limitation of CT images of the elbow as used in this study is the reduced depiction of humeral shaft length. Thus, the humeral shaft axis was estimated based on a length of 5 to 10 cm. Although, we recommend the standardization of humeral length in future studies, we found only negligible correlation coefficients ranging between -0.21 and -0.18 for correlations between the sagittal distance—measured as a surrogate for humeral lengths—and angle values. With regard to radiologic practice, it is a common goal to reduce the radiation dose which can be achieved by focusing on the specific area of interest like the elbow. Thus, as a clinician, it is important to be enable the estimation the humeral shaft axis based on a shorter humeral shaft as applied in this study. To validate this approach, future studies correlating the humeral shaft axis of different humeral shaft lengths with the axis of the whole humerus are needed.

Our study was performed on the mature distal humerus of healthy patients. Since our aim was to first describe normal angles, we did not include fractured distal humeri or collected data on movement deficits in the elbow. Thus, in future studies, the transfer of our data on clinical cases needs to be tested by including images of the injured distal humerus and collection of the range of motion in the elbow. Since our method was developed to analyze the unimpaired distal humerus, CT-scans of patients with arthrosis or osseous degenerations of the elbow cannot be measured using our method due to alterations of the bone caused by osteophytes or bone loss. Last, the inter-rater-reliability should be tested in future studies.

## Conclusion

We conclude that CT-imaging of the elbow adds an important feature to the understanding of the anatomy of the distal humerus. When underpinned with future studies, this might improve the standard radiologic evaluation of the distal humerus as it enables the assessment of the sagittal angle between different locations along the capitulum and trochlea in relation to the humeral shaft. Thus, besides the use of CT-imaging as a diagnostic tool in the detection of osseous defects, CT-based sagittal angle measurements might contribute to the explanation of elbow impairments and a more precise pre-operative planning and post-operative evaluation of osteosynthesis procedures at the distal humerus.

## Data Availability

The data that support the findings of this study are available from the corresponding author, Axel Przyklenk, upon reasonable request.
